# Length-dependent prediction of protein intrinsic disorder

**DOI:** 10.1186/1471-2105-7-208

**Published:** 2006-04-17

**Authors:** Kang Peng, Predrag Radivojac, Slobodan Vucetic, A Keith Dunker, Zoran Obradovic

**Affiliations:** 1 Center for Information Science and Technology, Temple University, Philadelphia, PA 19122, USA; 2 School of Informatics, Indiana University, Bloomington, IN 47408, USA; 3 Center for Computational Biology and Bioinformatics, Indiana University School of Medicine, Indianapolis, IN 46202, USA

## Abstract

**Background:**

Due to the functional importance of intrinsically disordered proteins or protein regions, prediction of intrinsic protein disorder from amino acid sequence has become an area of active research as witnessed in the 6th experiment on Critical Assessment of Techniques for Protein Structure Prediction (CASP6). Since the initial work by Romero *et al*. (Identifying disordered regions in proteins from amino acid sequences, IEEE Int. Conf. Neural Netw., 1997), our group has developed several predictors optimized for long disordered regions (>30 residues) with prediction accuracy exceeding 85%. However, these predictors are less successful on short disordered regions (≤30 residues). A probable cause is a length-dependent amino acid compositions and sequence properties of disordered regions.

**Results:**

We proposed two new predictor models, VSL2-M1 and VSL2-M2, to address this length-dependency problem in prediction of intrinsic protein disorder. These two predictors are similar to the original VSL1 predictor used in the CASP6 experiment. In both models, two specialized predictors were first built and optimized for short (≤30 residues) and long disordered regions (>30 residues), respectively. A meta predictor was then trained to integrate the specialized predictors into the final predictor model. As the 10-fold cross-validation results showed, the VSL2 predictors achieved well-balanced prediction accuracies of 81% on both short and long disordered regions. Comparisons over the VSL2 training dataset via 10-fold cross-validation and a blind-test set of unrelated recent PDB chains indicated that VSL2 predictors were significantly more accurate than several existing predictors of intrinsic protein disorder.

**Conclusion:**

The VSL2 predictors are applicable to disordered regions of any length and can accurately identify the short disordered regions that are often misclassified by our previous disorder predictors. The success of the VSL2 predictors further confirmed the previously observed differences in amino acid compositions and sequence properties between short and long disordered regions, and justified our approaches for modelling short and long disordered regions separately. The VSL2 predictors are freely accessible for non-commercial use at

## Background

Intrinsically disordered, or natively unfolded, proteins or protein regions do not fold into stable three dimensional (3-D) structures under physiological conditions; they instead exist as ensembles of non-cooperatively interchanging conformations in which the atom coordinates and backbone Ramachandran angles vary significantly over time with no specific equilibrium values [1-5]. Although lacking specific 3-D structures, many intrinsically disordered proteins/regions have been identified to carry out important biological functions [1-7]. It was further suggested that these functions indeed require disordered regions of flexible, dynamic conformations instead of rigid ordered regions [6]. Based on these findings, the protein trinity [7] or quartet [8] model was proposed as an alternative to the commonly accepted protein sequence-to-structure-to-function paradigm [9]. That is, native proteins or functional regions may exist in up to four forms – ordered, molten globule (collapsed disordered), pre-molten globule (extended disordered), and random coil-like (also extended disordered) – and functions may arise from any of these forms or from the transitions between them [7,8].

While the distinction between the molten globule and the two extended forms is fairly clear, the distinction between the pre-molten globule and the random coil-like forms is less certain. That is, several pre-molten globules have been observed to convert to molten globules by all-or-none transitions, suggesting that these partially folded conformations likely represent discrete forms [10]. On the other hand, in comparison to the random coil-like form, the pre-molten globule form is less extended, contains more (usually transient) secondary structure and also exhibits more evidence for hydrophobic clusters [11]. Furthermore, the pre-molten globule to random coil-like transition is featureless [10,12], suggesting that these two forms lie on a continuum with regard to the degree of backbone extension.

Due to their functional importance, it is essential to be able to reliably detect intrinsically disordered regions and such ability could have significant impact on a wide range of biomedical research. Although many experimental techniques exist [13,14], detecting intrinsic disorder might still be costly and time-consuming. Furthermore, it is often helpful to use more than one technique to completely characterize a disordered region since different methods could reveal different aspects of intrinsic disorder [13,14]. Alternatively, various computational algorithms have been developed for predicting intrinsically disordered regions from amino acid sequence [15-29]. The success of these predictors strongly supports the hypothesis that intrinsic disorder, like globular structure, is also encoded by the amino acid sequence [16]. Although not perfect, these predictors have been successfully used in many real-life applications, e.g. designing protein structure-function experiments [1,30], understanding the roles of disorder in cell-signalling and cancer-related proteins [31], improving prediction of protein phosphorylation sites [32], and improving the throughput of structural genomics pipelines [25,33]. Indeed, recent NMR experiments suggest that intrinsic disorder is a significant bottleneck in structural genomics efforts [33,34].

Most existing disorder predictors use a sliding window to map individual residues into a certain feature space, where a binary classifier can then be built to classify the residues as *disorder *or *order *using various machine learning algorithms. The features are usually extracted from the partial amino acid sequence within the window that directly reflects the compositional bias and unique properties that characterize intrinsic disorder. In some recent approaches, e.g. VL3-P [24] and DISOPRED2 [23], features are also derived from PSI-BLAST [35] generated profiles to incorporate evolutionary information. The improved performance of these approaches was consistent with the findings that intrinsically disordered regions have distinct evolutionary characteristics [36,37]. Recently, several novel algorithms have been proposed that do not require representing sequence with fixed number of features. For example, Dosztanyi *et al. *used the pairwise energy content estimated from amino acid composition to distinguish between folded and unfolded proteins/regions [27]. Yang *et al*. applied the regional order neural network (RONN) to estimate the disorder probability of a given sequence region based on its distances from a set of "prototype" disordered/ordered regions [28]. In another study [26], Coeytaux and Poupon developed a rule-based predictor for unfolded regions based on the amino acid propensity of being disordered and the distance to the nearest hydrophobic cluster.

Like the structural classification of ordered proteins, e.g. α-helix and β-sheet at the secondary structure level, and all α, all β, α/β and α+β classes at the tertiary structure level, we suggest that there are also several subtypes (flavors) of intrinsic disorder distinguished by amino acid compositions and sequence properties. It was first illustrated that long disordered regions characterized by different methods – X-ray diffraction, NMR, and circular dichroism (CD) – exhibited observable difference in amino acid compositions [38]. Using a supervised clustering procedure, it was discovered that there were at least three flavors (types) of long disorder, and three flavor-specific disorder predictors outperformed a global predictor (VL2) on the corresponding flavors [19]. On the other hand, amino acid compositions and sequence properties might also vary among disordered regions of different lengths, as indicated in our initial study of intrinsic disorder prediction [16]. This observation was further confirmed in a recent study [39] using much larger datasets, which clearly illustrated the significant differences between a set of disordered regions shorter than 11 residues and another set of disordered regions longer than 30 residues. However, due to difficulties in collecting disorder data, we did not pursue this issue further but instead focused on developing predictors specific for long disordered regions (>30 residues).

As revealed in the CASP5 experiment [40], most of the predictors that we tested were significantly less accurate on short disordered regions (≤30 residues) than on long disordered regions (>30 residues), with accuracies of 25–66% *versus* 75–95%, for short versus long regions of disorder, respectively. Given the length-dependent heterogeneity in amino acid composition, such a discrepancy is not surprising because these predictors were trained exclusively on long disordered regions. Another contributing factor might be the use of large sliding windows for feature construction (e.g. 41 residues) and output smoothing (e.g. 61 residues). In the first case, a large window could make residues from short disordered regions indistinguishable from those from ordered regions in the feature space, since most features were based on the local amino acid statistics within the window. In the second case, a large window would inevitably filter out many predicted short regions while improving prediction on long disordered and ordered regions.

Based on these findings, we developed a composite predictor called VSL1 [41] to address this length-dependent heterogeneity problem in disorder data. It consisted of three component predictors in a two-level architecture: at the first level there are two specialized predictors optimized for *long *(>30 residues) and *short *(≤30 residues) disordered regions, respectively; at the second level is a *meta *predictor for integrating the specialized predictors' outputs. As blind-test results in the latest CASP6 experiment showed, VSL1 significantly improved the prediction performance on short disordered regions, while retaining high accuracy on long disordered regions comparable to our previous long disorder predictors [41]. It also achieved the best prediction performance in almost all evaluation criteria used by the independent assessor [42].

In this report we describe two new predictors that are similar to VSL1. Although VSL2-M1 has identical architecture to VSL1, VSL2-M2 adopted a different approach (meta predictor) to integrate the specialized predictors. Another difference is that VSL2 component predictors were built as linear support vector machine (SVM) [43] instead of logistic regression models [44] in VSL1. Finally, the training dataset for VSL2 is slightly different from VSL1 by removing 8 mislabelled sequences. As the 10-fold cross-validation results showed, both VSL2 predictors achieved well-balanced prediction accuracies of about 81% on the two types of disordered regions, and were clearly superior to using either one of the specialized predictors alone. Comparisons over VSL2 training dataset via 10-fold cross-validation and a blind-test set of unrelated recent PDB chains indicated that VSL2 predictors were significantly more accurate than several existing predictors of intrinsic protein disorder. The results also showed that VSL2 had improved sensitivity over VSL1 but at the cost of reduced specificity.

## Implementation

### Datasets

#### Training dataset

A total of 1,327 non-redundant protein sequences, with pairwise sequence identity ≤25%, were used for VSL2 predictor training. These proteins were assembled from four other datasets: 153 sequences from DisProt (version 1.2) [45] with 160 long (>30 residues) and 28 short (≤30 residues) disordered regions, 511 PDB chains with 42 long and 929 short disordered regions [39], 290 completely folded PDB chains [39,46], and 373 recent PDB chains (released before June, 2004) with 15 long and 432 short disordered regions. His-tags and initial methionines were removed for further consideration from any applicable sequence.

In total there were 1,606 disordered regions with 34,911 residues and their length distribution is shown in Table 1. Of these disordered residues, about 72% came from 217 long disordered regions. While these long disordered regions were determined and validated by literature searches, the 1,389 short disordered regions were primarily identified as regions of missing electron density map in X-ray structures. In this study, we did not include the 483 very short disordered regions of 1–3 residues in either predictor training or accuracy estimation. Such short disordered regions are probably as likely to result from non-fitting structural environments as from their intrinsic sequences. Of the remaining 906 short disordered regions of 4–30 residues, 269 and 240 were at N- and C- termini, and contained 2,516 and 2,368 residues, respectively.

The training dataset contained a total of 406,342 ordered residues from the 1,327 sequences. For 320,339 of them we were able to extract their C_α _B-factors from PDB. These B-factors were first normalized to zero mean and unit variance after removing outliers, chain by chain, using a procedure by Smith *et al. *[46] The ordered residues were then assigned to two sets as *high-B-factor *(25,628 residues) and *low-B-factor *(294,711 residues) depending on whether their normalized B-factor values were higher than 2.0. The *high-B-factor *ordered residues were shown to have amino acid compositions and sequence properties similar to short disordered regions [39]. We therefore excluded *high-B-factor *ordered residues from predictor training since they might affect the training process. However, they were included in accuracy estimation.

#### Blind-test dataset

To facilitate performance comparison to other protein disorder predictors, we also constructed a blind-test dataset [see Additional File 1] of 1,304 recent PDB chains based on 2,101 PDB entries deposited between September 1, 2004 and December 21, 2005. All these structures (no protein/nucleic acid complexes) were determined by X-ray diffraction with resolution ≤2.5Å and R-value ≤0.25. For each chain in an entry, the sequence segments extracted from structural data, e.g. REMARK 465 (missing), ATOM, HETATM, and TER (terminal) records, were aligned to the corresponding SEQRES sequence. If any inconsistency or error was detected, the whole entry was discarded. In total 1,662 of the initial 2,101 entries were successfully processed, resulting in 3,967 chains of ≥40 residues. After removing His-tags or leading/trailing segments, disordered regions were then identified as residues of missing electron density based on the REMARK 465 record.

We then performed clustering analysis to remove redundant chains and chains similar to training sequences. The NCBI BLASTClust program [47] was applied to the union set of the new chains and the training sequences, with identity threshold of 25% (- S 25), minimal length coverage of 100% (-L 1.0) on only one sequence of a pair (-b F). If two chains fall into different clusters, they should have pairwise sequence identity <25%. Thus, the blind-test dataset was constructed by selecting one representative chain from each of the clusters that contained no training sequences, with the criteria as (a) highest resolution, (b) lowest R-value, and (c) most disordered residues. In total, the blind-test dataset contained 1,304 chains with 14,737 disordered residues and 318,431 ordered residues. The length distribution of these disordered regions is shown in Table 1. In this blind-test dataset about 19% of all disordered residues came from 54 long disordered regions, while in the VSL2 training dataset this proportion was about 72%.

### VSL2 architecture

**Figure 1:**
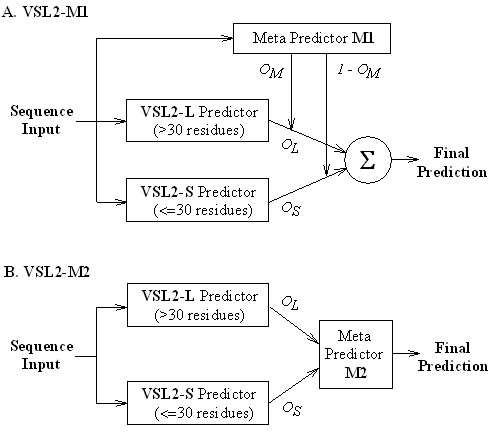
**VSL2 predictor architectures**. The final prediction for VSL2-M1 is calculated as *O*_*L *_× *O*_*M *_+ *O*_*S *_× (1 – *O*_*M*_), while for VSL2-M2 it is the output of meta predictor M2. The inputs for M2 are 2 × *W*_*in *_predictions by VSL2-L and VSL2-S for the neighbouring residues in a window of length *W*_*in*_. All component predictors are built using classification algorithms that approximate the posterior probability *p*(*c *= **1**|**x**), where **x **is the feature (input) vector and *c *is the class label.

Both VSL2-M1 and VSL2-M2 consist of three component predictors in two-level architectures (Figure 1). At the first level, there are two specialized predictors: a *short disorder predictor*, VSL2-S, for disordered regions of ≤30 residues, and a *long disorder predictor*, VSL2-L, for disordered regions of >30 residues. At the second level, there is a *meta predictor *that combines outputs of the two specialized predictors into the final prediction. All component predictors are built as binary classifiers that approximate the posterior class probability p(*c*=**1**|**x**), where **x **is the feature (input) vector and *c *is the class label. For the two specialized predictors, class **1 **data corresponds to *short disorder *or *long disorder*, while class **0 **data corresponds to *order*. Similar to previous disorder predictors, a set of features are extracted from the amino acid sequence and other related data using the standard sliding window approach.

Meta predictor M1 is trained independently of the two specialized predictors (Figure 1A). Its class **1**/**0 **data corresponds to residues that are either within or at most *(W*_*in *_– 1)/2 positions away from a *long/short *disordered region, where *W*_*in *_is an odd number. If the output *O*_*M *_is close to 1 (or 0), the current residue is more likely to be within or close to a long (or short) disordered region, and thus the specialized predictor VSL2-L (VSL2-S) should be given greater importance. If *O*_*M *_is close to 0.5, the current residue is more likely to be in an ordered region and both specialized predictors should contribute equally. Therefore, the final prediction can be calculated as *O*_*L *_× *O*_*M *_+ *O*_*S *_× (1 – *O*_*M*_), where *O*_*L *_and *O*_*S *_are outputs of VSL2-L and VSL2-S. For meta predictor M2 (Figure 1B), the inputs are 2 × *W*_*in *_predictions by VSL2-S and VSL2-L for residues within a symmetric window of length *W*_*in*_. Its output is the final disorder prediction.

### Feature construction

For the two specialized predictors and meta predictor M1, features were constructed for each residue based on an *input (sliding) window *of length *W*_*in *_(odd number) centred at the residue, where the value of *W*_*in *_is selected to maximize the prediction accuracy. The window was extended outside the N-/C- terminus by padding it with *(W*_*in*_*- *1)/2 special spacer characters. This approach is equivalent to the extra input per residue used in other protein structure predictors to indicate when the window spans the termini (e.g. [48-50]). In total, four sets of 54 features were calculated from amino acid sequences, sequence profiles, and secondary structure predictions.

The first set (**AA**) of 26 features was derived from local amino acid composition of the partial sequence within the input window, including the 20 amino acid frequencies, the spacer character frequency, the *K_2_-entropy *measure of local sequence complexity [51], the average net charge, the average hydrophobicity [52], the charge-hydrophobicity ratio [17], and the average flexibility index [53]. To incorporate evolutionary information, sequence profiles were generated by PSI-BLAST [35] searches (maximum 3 iterations) against the UniRef100 database [54]. As in the VL3-P predictor [24], the 20-column position-specific scoring matrix (**PSSM**) and the last 2 columns from the profile (i.e. *information per position *and *relative weight of gapless real matches to pseudocounts) *were averaged over the input window, resulting in the second set (PSSM) of 22 features. In addition, three secondary structure prediction scores by the PHDsec predictor [50] and another three by the PSIPRED predictor [48] were also included. While the PHDsec predictions were made without using multiple sequence alignment (evolutionary information), PSI-BLAST profiles were used for PSIPRED predictions. The prediction scores were then averaged over the input window to obtain another 6 features (**PHD **and **PSI**).

We applied feature selection using a permutation-test-based feature filter [55] and several other algorithms implemented in the WEKA data mining package [56]. Principal component analysis (PCA) was then performed to de-correlate the selected features and further reduce the sample dimensionality by keeping the variance at 95%.

### Predictor model

The component predictors were built as linear support vector machines (SVM) using the inner-product kernel [43]. A hyperplane in the feature space is learned from the training data to separate examples from the two classes *(disorder *and *order)*. By choosing the hyperplane that maximizes the margin, the resulting predictor could often achieve better generalization performance on out-of-sample data [43]. If the two classes cannot be well separated by a hyperplane in the original feature space, non-linear kernels (e.g. radius-based-function or RBF) can be used to map the data into a higher dimensional space where the two classes become separable. Due to its ability to handle complex, high-dimensional and noisy data, SVM has been widely used in various computational biology problems (for a review, please refer to [57]).

In this study we used the SVM^*light *^[58] implementation for building SVM predictors, since it is scalable and can handle very large datasets efficiently. In addition, a single-input logistic regression model was trained to calibrate the SVM output into posterior probability *p*(*c*=**1**|**x**) [59]. Given a set of training sequences, an embedded 5-fold cross-validation was performed to select the optimal parameter *C *which represents the trade-off between training error and margin [58]. A final SVM predictor can then be built with available sequences using the selected *C*. Note that the available training sequences here are not necessarily all 1,327 sequences in the training dataset, but could be 9/10 of them as in the 10-fold cross-validation procedure for accuracy estimation (see Performance evaluation below). To train the predictors, balanced datasets were always used by randomly sampling from available training sequences.

We also examined several other popular learning algorithms such as logistic regression models [44], feed-forward neural networks [60], neural network ensembles [61], and non-linear SVMs [43]. However, our results showed that none of them significantly outperformed linear SVMs.

### Output smoothing

As in our previous studies (e.g. [19] and [24]), a moving-average approach was applied to smooth the raw predictions to remove occasional misclassifications, based on the assumption that neighbouring residues often share the same structural property. In this approach, the final prediction for a given residue is calculated as the average of raw predictions for neighbouring residues within an *output window *of length *W*_*out *_(odd number) centred at that residue. Like the input window length *W*_*in*_*, W*_*out *_is also subject to optimization to maximize the prediction accuracy.

### Performance evaluation

A 10-fold cross-validation procedure was used to estimate the *out-of-sample *prediction accuracies. First, the training dataset ***D ***of 1,327 sequences were randomly divided into 10 disjoint subsets ***D***_***1***_, ***D***_***2***_, ..., ***D***_***10 ***_of roughly equal sizes. Second, in the *i*-th fold, *i *= 1,2,...,10, a VSL2 predictor was built with sequences in ***D – D***_***i ***_only and then applied to the sequences in ***D***_***i***_. After the procedure is complete, predictions for all sequences from ***D ***can be obtained. In this way, the prediction for any sequence is always made using a predictor trained without that sequence. Finally, prediction accuracies (see below) can be estimated using predictions for all 1,327 sequences to evaluate the performance.

In the *i*-th fold, *i *= 1,2,...,10, component predictors were first trained with all *applicable *sequences in ***D – D***_***i ***_only. The optimal SVM parameter *C *for each component predictor was selected using "embedded" 5-fold cross-validation as described above (see Predictor model). For specialized predictors VSL2-S and VSL2-L, class **1 **examples were drawn from short and long disordered regions respectively, and class **0 **examples were drawn from *low-B-factor *ordered regions (see Datasets). The training data for meta predictor was constructed as described above (see VSL2 architecture), also using sequences from ***D – D*_*i *_**only. Once the component predictors were built, the VSL2 predictor was assembled and applied to sequences from ***D***_***i***_.

For a given predictor, the overall accuracy *(ACC) *is measured as the average *of sensitivity (SN) *and *specificity (SP)*, where the *sensitivity*, or *true positive rate*, is the percentage of class **1** examples correctly predicted, and the *specificity*, or *true negative rate*, is the percentage of class **0 **examples correctly predicted, using a certain decision threshold (typically 0.5). Compared to other performance measures, such as *Q2 *score (percentage of all correctly predicted residues) [62] and *Matthews' correlation coefficient *[63], the *ACC *measure is more suitable for datasets of imbalanced class proportions. In such a case, a random predictor or a trivial predictor that assigns all examples to one class will have an overall accuracy of 50%.

For both specialized predictors and the final predictors, three sensitivities – *SN*_*S*_, *SN*_*L *_and *SN *– are reported for *short*, *long *and *all *disordered regions, respectively. Accordingly, the overall accuracies were calculated as *ACC*_*L *_= (*SN*_*L *_+ *SP*)/2 for VSL2-L, *ACC *= (*SN*_*S *_+ *SP*)/2 for VSL2-S, and *ACC *= (*SN + SP*)/2 for the final predictors. For meta predictors, we did not explicitly estimate their accuracies but instead used the accuracies of the corresponding final predictors for performance evaluation.

Unless explicitly specified, the accuracies reported are *per-chain *accuracies, i.e. *SN*_*S*_, *SN*_*L*_, *SN *and *SP *were first calculated on each individual chain (if applicable) and then averaged over chains. Since about 72% of the disordered residues in our training data came from long disordered regions, *per-residue *accuracies *(SN *and *ACC) *could be dominated by the performance on long disordered regions. Even for the long disorder predictor VSL2-L, its *per-residue *accuracy *(SN*_*L*_*) *could be biased toward certain extremely long disordered regions. Therefore, we chose *per-chain *accuracies for model selection and predictor comparison but also reported *per-residue *accuracies. Note that among the *per-chain *accuracies, *SN *could be higher than both *SN*_*S *_and *SN*_*L *_because some chains may have both short and long disordered regions and the three sensitivities were averaged over different subsets of chains.

In addition to the accuracies calculated with the default threshold 0.5, we also plotted the receiver operating characteristic (ROC) curve and calculated the area under the ROC curve *(AUC) *[64]. The ROC curve is a plot of sensitivity against (1 – specificity), usually calculated at different decision thresholds. That is, each point on the ROC curve corresponds to a specific threshold used. The area under the ROC curve *(AUC) *is known to be a useful measure of overall predictor quality, with a value of 100 for a perfect predictor and 50 for a random predictor. Note that ROC curves could also be *per-chain *or *per-residue *version, depending on the type of sensitivity and specificity used.

Finally, a bootstrap [65] procedure was used to estimate the standard error for each accuracy measure discussed above. More specifically, 5,000 bootstrap replicated samples were drawn from of the 1,327 sequences with replacement, and the accuracies were calculated on each bootstrap sample. The standard errors were then reported as one standard deviation of the results obtained over the 5,000 runs.

## Results and discussion

### Length dependent amino acid compositions

**Figure 2:**
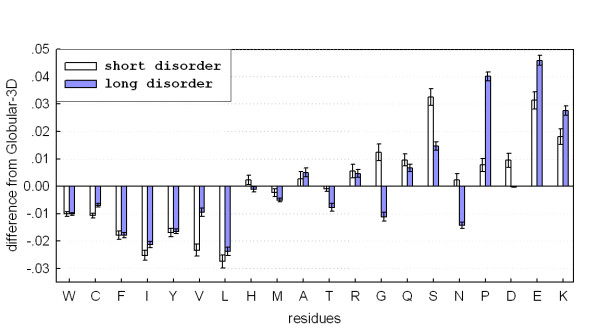
**Comparison of amino acid compositions between short and long disordered regions**. The **y**-axis represents the difference in amino acid compositions (fractions) from a reference dataset of *ordered *proteins, Globular-3D. The error bars correspond to one standard deviation estimated using 5,000 bootstrap samples. His-tags and initial methionines were not counted.

The amino acid compositions of short (4–30 residues) and long (>30 residues) disordered regions were compared to the composition of a reference ordered dataset, Globular-3D [18]. As shown in Figure 2, both types of disordered regions exhibit similar overall compositional bias that characterizes intrinsic protein disorder [38], i.e. depletion of the typically buried W, C, F, I, Y, V and L and enrichment of the typically exposed K, E, P, S, Q and R. However, there were also some significant differences. Short disordered regions are more depleted in C, I, V and L, while long disordered regions are more enriched in K, E and P but are less enriched in Q and S. In addition, long disordered regions are depleted in G and N, while short disordered regions are enriched in G and D.

### Specialized predictors – window lengths

The optimal *W*_*in*_*/W*_*out *_combination was selected from all 169 possible pairs of *W*_*in *_∈ {11, 15, 21, 25, ..., 71} and *W*_*out *_∈ {1, 5, 11, 15, ...,61}, to maximize the overall accuracy, i.e. the average of sensitivity and specificity. Using all 54 features, the two predictors were built as linear SVMs with parameter C, which represents the trade-off between training error and margin [58], set to 0.5 and 1, respectively. In this way, the optimal *W*_*in*_*/W*_*out *_combination for the short disorder predictor VSL2-S was selected as 15/5, considerably smaller than 41/31 for the long disorder predictor VSL2-L. As shown in Table 2, with the selected *W*_*in*_*/W*_*out *_values, VSL2-S achieved much higher accuracy on short disordered regions than on long ones *(SN*_*S*_*:*82.0 ± 1.1% *versus *44.1 ± 1.8%), while VSL2-L was significantly more accurate on long disordered regions but with a smaller difference *(SN*_*L*_: 82.1 ± 2.2% *versus *70.7 ± 1.9%). On ordered regions, VSL2-L was more accurate than VSL2-S *(SP: *87.3 ± 0.5% *versus *81.5 ± 0.3%).

Also shown in Table 2 are prediction accuracies for several non-optimal *W*_*in*_*/W*_*out *_values. A general conclusion from these results is that smaller windows are necessary for predicting short disorder. Using a large input window (W_*in*_), it might be more difficult to distinguish short disorder residues from order residues in the feature space, since most features were based on the local amino acid composition within the window. In such a case, too many neighbouring order residues may be included in the window and the compositional bias information necessary for predicting short disorder would be weakened. Similarly, a large output window *(W*_*out*_*) *would inevitably filter out many predicted short regions while improving prediction on long disordered and ordered regions.

Table 2 also suggests that window length alone could not account for accuracy discrepancy between short and long disorder by either VSL2-S or VSL2-L. When *W*_*out *_was increased from 5 to 31, VSL2-S improved only slightly on long disordered regions (71.3%) but deteriorated significantly on short disordered regions (56.5%). Increasing both *W*_*in *_and *W*_*out *_did not significantly improve VSL2-S accuracy on long disordered regions (74.3%), either. Similarly, when *W*_*out *_was decreased from 31 to 5, VSL2-L still performed poorly on short disordered regions (50.1%) and it was slightly less accurate on long disordered regions (80.9%). Decreasing both *W*_*in *_and *W*_*out *_significantly improved VSL2-L accuracy on short disordered regions (70.8%), but it was still much lower than VSL2-S. These results indicate that the difference in amino acid compositions between short and disordered regions is significant.

### Specialized predictors – feature selection

Once the optimal window lengths were selected, we performed feature selection for both specialized predictors using a permutation-test-based feature filter [55] and several other algorithms implemented in the WEKA data mining package [56]. However, no improvements in prediction accuracy were observed for either of the predictors. If removing about half (27) of the features, the prediction accuracies of both predictors would decrease by 1–2%. Such phenomenon might be explained by the relatively high correlations among features. Since the principal component analysis (PCA) was always performed (keeping 95% variance) before predictor training, such correlations would be removed and are unlikely to cause problems. Furthermore, the number of available training examples (residues) is sufficiently large compared to the number of features (54) and the overfitting problem might be less likely to occur. Therefore, we did not exclude any features but used PCA only to reduce the dimensionality.

In Table 3 we list the 20 features ranked on top according to their permutation test Z-scores [55]. The absolute value of such a Z-score reflects the relevance of a given feature, while the sign indicates if the feature is positively/negatively correlated with the target variable (i.e. **1 **for disorder and **0 **for order). The top feature for VSL2-S is the spacer frequency *(freq_spacer)*, which indicates if a residue is close to a terminus (see Feature construction). Its high positive Z-score is consistent with the fact that many short disordered regions in our dataset were at termini. Another observation from Table 3 is that many of the features were PSI-BLAST profile based (PSSM_*) but only few were amino acid frequencies (freq_*). This suggests that the profile based features with evolutionary information indeed have higher discriminative power in prediction of both short and long disorder. Note that hydrophilic residues were more conserved in both short and long disordered regions as compared to ordered, while hydrophobic residues were more conserved in ordered regions.

In total there are 12 features in common between the two subsets selected for VSL2-S and VSL2-L, but they typically had different ranks. Among these features, *K*_*2*_*-entropy *(local sequence complexity) [51], *flexibility *[53] and *hydrophobicity *[52] are known indicators of protein disorder, *PSSM_D, PSSM_E, PSSM_K, PSSM_P, PSSM_Q *and *PSSM_S *correspond to *disorder-promoting *residues [18], and *PSSM_I *corresponds to an *order-promoting *residue [18]. Another two shared features are *PSI_C *and *PHD_L *derived from the secondary structure predictions for coils (loops) by the PHDsec and PSIPRED predictors, respectively. The different ranks for VSL2-S and VSL2-L of certain features might be directly connected with the compositional difference between short and long disorder (Figure 2), e.g. *PSSM_P *and *PSSM_K*.

### Specialized predictors – choice of learning algorithm

We also examined other learning algorithms for component predictors, including a logistic regression model [44], a feed-forward neural network of single hidden layer (5 hidden nodes) [60], a bagging ensemble of 10 neural networks [61], and a non-linear SVM with radius-based-function (RBF) kernel [43]. The accuracies reported in Table 4 were estimated using the 10-fold cross-validation procedure (see Performance evaluation). The optimal model parameters, e.g. *C *for linear SVM and *C *and *gamma *for RBF SVM, were selected using embedded 5-fold cross-validation (see Predictor model) independently for each fold of the 10-fold cross-validation procedure. We examined *C *from {2^-2^, 2^-1^, 1, 2, 4, 8} for linear SVMs, and *C* and *gamma* from {2^-2^, 2^-1^, 1, 2, 4, 8} × {2^-6^, 2^-5^, 2^-4^, 2^-3^, 2^-2^, 2^-1^, 1} for RBF SVMs. It turned out that identical parameters were selected in most of the 10 folds for accuracy estimation (results not shown). These parameters were reported in Table 4 and used for building the final VSL2 predictors for blind-test comparison with other predictors.

As shown in Table 4, the algorithms examined had similar prediction accuracies as linear SVM but none of them outperformed it significantly. The small differences between linear and nonlinear models are consistent with the observations in our previous studies [19,24], which reflected the linear nature of the disorder prediction problem. Since it has been shown that SVM often have better generalization performance than other learning algorithms, in the subsequent analyses we report results for linear SVM only.

### Combining specialized predictors

The VSL2-S and VSL2-L predictors were then integrated into the composite VSL2-M1 and VSL2-M2 predictors using two different meta predictors, M1 and M2, respectively. Meta predictor M1 was trained independently of VSL2-S and VSL2-L using the same set of 54 features. Inputs of meta predictor M2 were *W*_*in *_neighbouring predictions from both VSL2-S and VSL2-L, and its output was the final prediction of VSL2-M2. Both M1 and M2 were built as linear SVM *(C = *1). Using the 10-fold cross-validation procedure (see Performance evaluation), the optimal *W*_*in*_*/W*_*out *_values were selected as 61/1 and 31/1 for M1 and M2, respectively.

In Table 5 we show prediction accuracies of the two composite VSL2 predictors, as well as the two specialized predictors. Among the four predictors, VSL2-M1 achieved the highest sensitivity *(SN) *of 82.3 ± 1.1% and overall accuracy (*ACC*) of 81.6 ± 0.5%, and VSL-M2 had very similar performance. Although the short disorder predictor VSL2-S also had relatively high *SN *of 79.8 ± 1.0% and *ACC *of 80.7 ± 0.5%, it was significantly less accurate (by 11%) on long disordered regions. On the other hand, both VSL2-M1 and VSL2-M2 had well-balanced *per-chain *accuracies *(SN*_*S *_and *SN*_*L*_*) *of >81% on short and long disordered regions, which are comparable to the accuracies by the corresponding specialized predictors. On ordered regions, both VSL2-M1 and VSL2-M2 were significantly less accurate than VSL2-L but still comparable to VSL2-S.

### Length-dependent prediction accuracy

**Figure 3:**
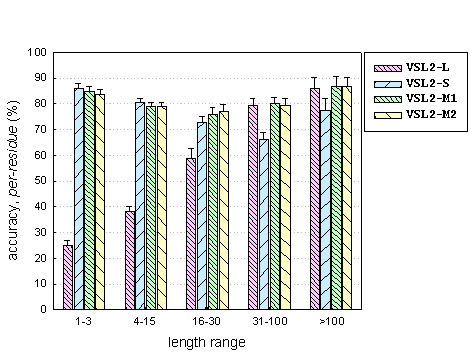
**Length-dependent prediction accuracies**. *Per-residue *accuracies (*sensitivities*) are reported on disordered regions from different length ranges.

To better characterize the predictor performance, we further divided the disordered regions into five length groups of 1–3, 4–15, 16–30, 30–100, and >100 residues and examined the *per-residue *accuracy (sensitivity) on each group separately. Note that short disordered regions of 1–3 residues were not used in predictor training or accuracy estimation. As shown in Figure 3, VSL2-L performed poorly on very short disordered regions, and exhibited a monotonic increase in accuracy as the disordered region length increases. VSL2-S had the highest accuracies on short disordered regions of 1–3 and 4- 15 residues, but was significantly less accurate on long disordered regions than VSL2-L. On the other hand, both VSL2-M1 and VSL2-M2 achieved almost uniform prediction accuracies over different length groups. In every length group the two composite predictors achieved similar or even higher accuracies than the corresponding specialized predictor.

Another observation from Figure 3 is that VSL2-M1 and VSL2-M2 were less successful on disordered regions of 16–30 residues than on those from the other length groups. One possible explanation is that the threshold of 30 for partitioning disordered regions into *short *and *long *is artificial [6], and therefore is not necessarily optimal. It is also likely that the amino acid compositions of disordered regions of different lengths form a continuum. Therefore, partitioning disordered regions into *short *and *long *by a single length threshold might not be the most appropriate approach. However, it could be helpful to introduce a third length group of *medium *disordered regions, but a larger dataset would be necessary. A better approach might be applying a competition procedure developed previously [19] to further improve the initial partitioning obtained by the length threshold.

Since VSL2-M1 was slightly more accurate than VSL2-M2, it was used exclusively in the following analyses. For brevity, we will refer to it as "VSL2".

### Importance of computationally expensive features

As shown in Table 3, most of the top-ranked features for the specialized predictors were based on PSI-BLAST profiles (PSSM_*) or secondary structure predictions *(PSI_C *and *PHD_L)*. However, obtaining the PSI-BLAST profile and/or PSIPRED prediction is time-consuming due to the need for searching against large sequence databases. On a workstation with a 3GHz Pentium^® ^4 processor and 2GB memory, it took 3.3 minutes on average for a single PSI-BLAST search consisting of 3 iterations against the UniRef100 database [54] (May 2004 release, 1, 115,083 sequences, required by PSIPRED). Clearly, this may result in a computational bottleneck if VSL2 is used in genomic-scale studies. Therefore, it is important to examine the accuracy tradeoffs if these computational expensive features are not included.

Table 6 compares the prediction accuracies of VSL2 and its 7 variants which use different combinations of the four feature sets **AA, PHD, PSI **and **PSSM**. The amino acid composition based (**AA**) features were always included since it can be calculated directly from the sequence. Note that VSL2 used all four feature sets. The baseline predictor using only **AA **features (Table 6, 1^st ^row) will be denoted as "VSL2B" in the following discussions. Compared to VSL2B, VSL2 significantly improved the sensitivities (*SN*, *SN*_*S *_and *SN*_*L*_*) *by 4. 1–5.5% and the specificity *(SP) *by 1.1%. If using only **AA **and **PSSM **features (Table 6, 5^th ^row), the resulting predictor achieved accuracies similar to VSL2, with only 1.3% lower in *SN *and 0.6% lower in *SP – *we will denote it as "VSL2P". The **PHD **features could be calculated relatively efficiently (4.8 seconds per sequence on average) since we did not use multiple sequence alignment for PHDsec predictions. As expected, it was less informative than the **PSI **features (Table 6, 2^nd ^and 3^rd ^rows).

Although the baseline predictor VSL2B was 3% and 2.1% inferior to VSL2 and VSL2P, respectively, in the overall accuracy (*ACC*), it also achieved relatively balanced accuracies on short and long disordered regions, and was more accurate than several previous disorder predictors (see below). Therefore it may still be useful in some genome-scale studies. For example, VSL2B can be applied first to a whole proteome to identify a smaller sequence subset of interest, and then the more accurate but time-consuming VSL2 (or VSL2P) can be used to further improve the results.

### Prediction on high-B-factor ordered regions

As shown by Radivojac *et al. *[39], *high-B-factor *ordered regions were similar to, but also had some significant differences from, short disordered regions in terms of amino acid compositions and sequence properties. In addition, they could be predicted fairly accurately from amino acid sequence using features similar to those for disorder prediction. We therefore excluded *high-B-factor *ordered regions from VSL2 predictor training and examined the prediction accuracy on these regions. Indeed, VSL2 had a much higher false positive error rate on the *high-B-factor *residues than on the *low-B-factor *residues (43% *versus *17%). On the outliers (i.e. residues with extremely high B-factors, detected during the normalization procedure [46]) the false positive rate was even higher (51%). However, due to the small proportion (8%) of *high-B-factor *residues, the overall false positive rate was only slightly higher (19%) than on *low-B-factor *residues. Note that the predictions used were obtained using the 10-fold cross-validation procedure (see Performance evaluation); the prediction for any sequence was made using a predictor trained without this sequence.

### Representative predictions

**Figure 4:**
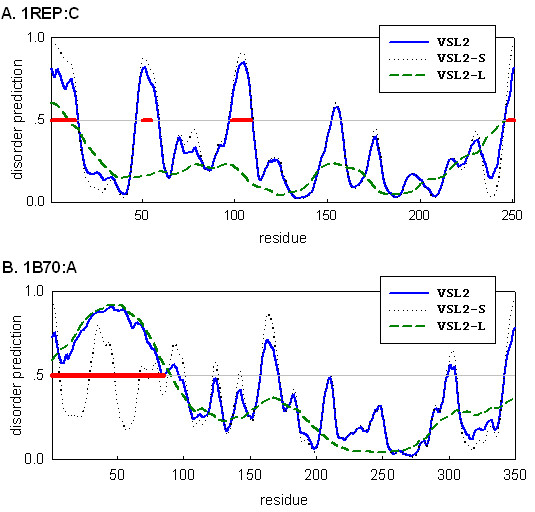
**Representative predictions on two PDB chains**. (A) 1REP:C with four short disordered regions at residue 1–14, 50–55, 98–109, and 247–251. (B) 1B70:A with a long disordered region at residue 1–85. These disordered regions are marked as thick line segments. Residues with predictions above 0.5 are interpreted as predicted disordered.

In Figure 4 we show representative predictions on two PDB chains: (A) 1REP:C with four short disordered regions at residues 1–14, 50–55, 98–109, and 247–251; (B) 1B70:A with a long disordered region at residues 1–85. The short disorder predictor VSL2-S successfully detected all four short disordered regions in 1 REP:C, while the long disorder predictor VSL2-L predicted only part of the two terminal regions and completely missed the two internal regions (Figure 4A). On the other hand, VSL2-L correctly identified the whole long disordered region from 1B70:A, while VSL2-S predicted only 37 of the 85 residues as disordered (Figure 4B).

It can be observed from Figure 4 that the final VSL2 prediction is more similar to VSL2-S over short disordered and ordered regions, while it is more similar to VSL2-L over long disordered regions. In Figure 4A, VSL2 prediction was almost indiscernible from VSL2-S prediction along the whole sequence. In Figure 4B, VSL2 prediction was similar to VSL2-L prediction over the first 85 long disordered residues, and then started resembling VSL2-S prediction over the remaining region. This illustrates the effectiveness of the meta predictor. It also partly explains the relatively low specificity (81.0%) of VSL2 (Table 5), since VSL2-S tends to predict many false short disordered regions over ordered regions (Figure 4B).

### Comparison to a global predictor

For comparison, we also built a global disorder predictor as a single binary classifier. It was trained in the same way as the two specialized predictors, but with disorder residues equally sampled from all disordered regions. The optimal *W*_*in*_*/W*_*out *_values were determined as *W*_*in*_*/W*_*out*_*= *21/11, right between the optimal values for VSL2-S and VSL2-L (Table 2). Its accuracies were estimated as *SN*_*S*_*= *76.9 ± 1.4%, *SN*_*L*_*= *76.3 ± 1.9% and *SP = *83.9 ± 0.4% on short disorder, long disorder and order, respectively. Although having well-balanced performance on the two types of disorder, the global predictor was significantly less accurate than VSL2 (Table 5).

### Comparison to previous disorder predictors

In this section we compare three VSL2 predictors (VSL2B, VSL2P and VSL2) to six previously developed protein disorder predictors over both VSL2 training dataset via 10-fold cross-validation and a blind-test set of 1,304 unrelated recent PDB chains. These predictors included three of our previous long disorder predictors, VL-XT [18], VL3-E [24] and VSL1 [41], as well as three predictors developed by other groups, i.e. DisEMBL (remark 465 definition only) [21], RONN [28] and DISOPRED2 [23]. There are several other disorder predictors were not included for different reasons. For example, PreLink [26] does not provide numeric predictions, Foldlndex^© ^[66] and GlobPlot [20] do not provide predictions for all residues, while IUPred predictor [27] provides separate predictions for short disordered, long disordered, and structured (ordered) regions, thus makes it difficult to compare.

#### Comparison over VSL2 training dataset via 10-fold cross-validation

**Figure 5:**
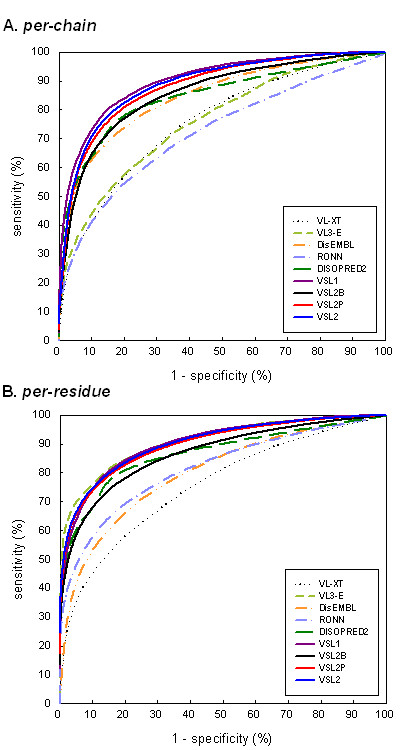
**Comparison of receiver operating characteristic (ROC) curves**. The ROC curves were plotted using (A) *per-chain *and (B) *per-residue *accuracies, by varying the decision thresholds from 0 to 1 in increments of 0.001. The corresponding *AUC *values were approximated using the *trapezoid rule *and reported in Table 7.

Predictions by the three VSL2 predictors were made via the 10-fold cross-validation procedure, i.e. the prediction for any sequence was made with a predictor trained without that sequence, while all other predictors were applied to the 1,327 sequences directly. RONN predictions were obtained from its website, while DisEMBL and DISOPRED2 were downloaded and run locally. Figure 5 compares both *per-chain *and *per-residue *ROC curves plotted by varying the decision threshold in increments of 0.001. The corresponding AUC values were approximated using the *trapezoid rule *and reported in Table 7. Also shown in Table 7 are prediction accuracies calculated with default thresholds. A threshold of 0.5 was used for all six predictors from our group.

As in Figure 5A and Table 7a, VSL2P, VSL2 and VSL1 had very close *per-chain *ROC curves with similar *AUC *values of 88.0 ± 0.6, 89.2 ± 0.5 and 89.9 ± 0.6, which were significantly higher than those of other predictors. Overall VSL2 was slightly less accurate than VSL1, with *ACC *of 81.6 ± 0.5% *versus *82.2 ± 0.6%. However, VSL2 had significantly higher sensitivities on disordered regions with differences >3%, while its specificity was 4.3% lower (Table 7a). DisEMBL and DISOPRED2 exhibited very high specificity coupled with low sensitivity, which is not surprising since they were tuned to generate very few false positives. As the ROC curves (Figure 5) suggested, VSL2 could also achieve similar trade-off between specificity and sensitivity by adjusting its decision threshold.

VL3-E was the most accurate on long disordered regions with a *per-chain *sensitivity (*SN*_*L*_) of 82.5 ± 2.2% (Table 7a) and a *per-residue *sensitivity of 85.7 ± 2.7% (Table 7b). Although VSL2 also achieved similar accuracy on long disordered regions, its specificities *SP *were more than 10% lower than VL3-E. On the other hand, VL3-E was the least accurate on short disordered regions. Since about 72% of the disordered residues in our dataset came from long disordered regions, it is not surprising that *per-residue SN *of VL3-E was much higher than its *per-chain SN *(70.4 ± 3.1% *versus *38.7 ± 1.6%). Furthermore, VL3-E had the highest *AUC *value of 90.9 ± 1.0 among the *per-residue *ROC curves, but it was just slightly higher than those of VSL2 and VSL1 (Figure 5B). RONN also exhibited much higher accuracy on long disordered regions, possibly due to the exclusive use of disordered regions longer than 20 residues in training [28].

#### Comparison over a blind-test set

The non-redundant blind-test set [see Additional File 1] consisted of 1,304 recent PDB chains that were unrelated to any sequence for VSL2 training. In Table 8 we show both prediction accuracies and areas under ROC curves *(AUC) *for the nine predictors. Note that the three VSL2 predictors were re-trained using *all *1,327 training sequences for this comparison. We did not use the 791 very short disordered regions of 1–3 residues in estimating accuracies *(SN*_*S *_and *SN) *since such short regions of disorder could result from many causes other than intrinsic sequence features. However, VSL2 predicted a higher proportion of residues as disordered in these regions than in those longer than 3 residues (79.8% *versus *74.7%).

Comparing Table 7 and Table 8, the predictor rankings by *per-chain ACC/AUC *were very similar, with VSL1, VSL2 and VSL2P constantly ranked on top. The main difference is that based on *per-residue ACC/AUC *VL3-E moved from the top in Table 7b to the 8^ th^/7^th ^place in Table 8b. It is evident from Table 7 and Table 8 that *SN*_*L*_, accuracy of all predictors dropped significantly with differences ranging from 9.2% (DisEMBL) to 26.5% (VL3-E), while *SN*_*S *_accuracy on short disordered regions and *SP *accuracy on ordered regions were less affected for most predictors. Overall, VL3-E and RONN were the two most affected predictors, with performance drops of 17.3% and 9.0% in* per-residue ACC*, and 15.0 and 10.2 in* per-residue AUC*, respectively.

By examining VSL2 prediction on the 54 long disordered regions, we observed that the prediction accuracy was >80% for 34 regions, 60–80% for 5 regions, and <45% for the remaining 15 regions. We attempted to obtain further information regarding these 15 regions with low prediction accuracies. However, at the time of this writing only five of them had been published as indicated by their PDB records and literature search. Based on the available publications, we examined three regions in more detail, namely 2A6T:B (residues 1–34, accuracy 20.6%) [67], 1Y44:A (residues 159–196, accuracy 21.1%) [68], and 1YYH:B (residues 1–54, accuracy 37.0%) [69]. The results demonstrate the uncertainties in labelling of long regions of missing electron density and may help explain the low prediction accuracies on some of these regions.

PDB entry 2A6T contains crystal structure of the 266-residue N-terminal of Dcp2 protein from *S. pombe *[67]. It forms a dimer of two identical chains in the asymmetric unit. However, the regions of missing electron density are not exactly the same for the two chains. Residues 1–34 are missing in chain B but are visible with ordered structure in chain A. As She *et al. *[67] suggested, this missing region might be caused by crystal packing. In addition, visible parts of the N-terminal domain of chain B seem to be superimposed well with the equivalent parts of chain A. Similarly, PDB entry 1Y44 also contains a dimer of two identical chains but with different missing regions [68]. Residues 159–196 missing in chain A correspond to the long "flexible arm" which is clearly folded in chain B. Thus, these two regions might be intrinsically *ordered *but missing in the electron density maps due to other reasons, which explains the low prediction accuracies on them. On the other hand, it is also possible that a sequence region that is intrinsically disordered as a monomer might undergo disorder-to-order transition upon multimer formation [70]. Clearly, both scenarios raise a unique question in data labelling.

We also examined PDB entry 1YYH (chain B) which contains crystal structure of the human Notch 1 ankyrin domain [69]. This domain consists of seven ANK (ankyrin) repeats and also forms a dimer of two identical chains, with almost identical missing density region (residues 1–51 for chain A and 1–54 for chain B). This region is considered to be a putative ANK repeat, i.e. the 1st repeat in the domain, which is comprised of two predicted a-helices connected by an unusually long loop of 16 residues [69] (note that the current Jpred [71] and PSIPRED [48] predictors both predicted another α-helix inside this region, but at low confidence). It was further suggested that the whole repeat is only partially folded due to the large number of charged or polar residues in the long loop [72].

**Figure 6:**
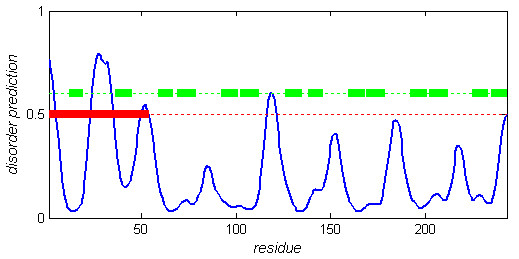
**VSL2 prediction on PDB chain 1 YYH:B**. VSL2 prediction (disorder probability) is plotted in blue sold line. Residues with predictions above 0.5 are interpreted as predicted disordered. The long region of missing electron density (residues 1–54) is marked as thick red segment. The fourteen short green segments correspond to the α-helices in the seven ANK repeats (two helices for each repeat).

In Figure 6, we plot VSL2 prediction score (disorder probability) on 1YYH:B. In the missing density region (residues 1–54), VSL2 predicted two ordered regions (residues 5–22 and 35–49) that roughly correspond to the two predicted α-helices. It also predicted one short disordered region (residues 23–34) that covers most of the loop region between the two predicted α-helices. In total, 20 (37.0%) of the 54 residues of this missing density region were predicted to be disordered. In the same figure we also marked the 6 pairs of α-helices of the other 6 ANK repeats in the human Notch 1 ankyrin domain [69]. Interestingly, there are 6 peaks (from the 3rd one) corresponding to the 6 beta turn regions between each pair of neighbouring ANK repeats.

In summary, just as in the two recent CASP experiments [42,62], disordered regions in our blind-test dataset were identified from missing electron densities in X-ray structures using an automatic procedure. As we discuss above, some of these regions are likely to be incorrectly labelled since missing density regions can be caused by reasons other than intrinsic disorder. For example, crystal packing irregularities can lead to missing electron density. Also, long regions of missing density can be structured, "wobbly domains" [3,13,73] that move as rigid bodies and thus fail to scatter X-rays coherently. Further experiments (e.g. protease digestion and NMR) as well as computational analysis can be used to identify such cases. Another situation is that a long missing density region is partially folded (or unfolded); as in the case of PDB:1YYH[69], labelling the whole region as disordered resulted in poor accuracy even though the predictor actually predicts the real situation. Finally, as in the case of PDB:2A6T[67] and PDB:1Y44[68], protein complexation can result in ambiguity in labelling, i.e. identical chains may have different missing density regions. From the beginning we have been aware of the possibility of mislabelling when disorder is identified only from missing coordinates in X-ray structures [16], and for this reason we have tried to corroborate our main findings with disorder identified by other methods such as CD and NMR [13,18,38,73].

## Conclusion

In this study we addressed the length-dependency problem in prediction of intrinsic protein disorder, i.e. that the amino acid compositions and sequence properties may vary among disordered regions of different lengths. As already observed in several previous studies, such length-dependency could result in inferior predictions if it is not taken into account explicitly. Therefore, we proposed two new predictor models, VSL2-M1 and VSL2-M2, in which specialized predictors were built for short disordered regions (≤30 residues) and long disordered regions (>30 residues) and then integrated via the meta predictor. The results suggested that the proposed VSL2 predictors achieved well-balanced accuracies on both short and long disordered regions and were significantly more accurate than several previous intrinsic protein disorder predictors.

The success of VSL2 predictors can be attributed to (a) the enlarged training data containing both long disordered regions (>30 residues) and short disordered regions (≤30 residues), and (b) the architecture for explicitly exploiting the data heterogeneity, or length dependency in the amino acid compositions and sequence properties of disordered regions. Under the two-level architecture, the specialized predictors, VSL2-S and VSL2-L, could be optimized separately on more homogeneous data. Both meta predictors proved effective in combining the two specialized predictors with comparable or even improved performance on both short and long disordered regions. These results further confirmed the previously observed differences between short and long disordered regions and justified our approach to model them separately.

There are several directions for further improving the VSL2 predictors. While the prediction performance of the long disorder predictor VSL2-L seems to be approaching its limit, there might be room for improving the short disorder predictor VSL2-S, e.g., by reducing its false positive rate. To achieve this, we will examine the relationships between short disordered regions and high B-factor ordered regions, oligomer interfaces and crystal contacts in more detail. Special treatment of terminal disordered regions needs to be developed, while techniques for denoising the training data and improving data representation should also be employed. Finally, new approaches that incorporate long-range interactions and other information should also be investigated.

## Availability and requirements

The VSL2 (VSL2-M1) predictors are freely accessible for non-commercial use via the web site at . This site provides web interface to two VSL2 variants, VSL2B and VSL2P (see Importance of computationally expensive features). Due to available computational resources, the number of predictions that can be provided per IP address per day is limited.

One can also download the VSL2 predictor package (Java executable) from the same website at . Note that the secondary structure predictors (PHDsec and PSIPRED), PSI-BLAST and sequence database are not included in this package, but should be downloaded separately from related websites. For detailed installation instructions, please refer to the README file in the package or a web page at .

## Authors' contributions

KP carried out the predictor and website development and drafted the manuscript. PR prepared the dataset for predictor training. PR and SV participated in predictor development and evaluation. ZO and AKD conceived and supervised the project. All authors have read and approved the final manuscript.

## Supplementary Material

Additional File 1 Disordered regions are marked as "#<starting position>-<ending position>" in FASTA headers. If a chain has no disordered region, its FASTA header will contain the PDB entry and chain ID only. Click here for file

## Figures and Tables

**Table 1:** Length distributions of disordered regions. VSL2 training dataset contained 1,327 sequences, while the blind-test dataset had 1,304 recent PDB chains that were unrelated to any training sequences. Both datasets were non-redundant with pairwise identity ≤25%.

	**VSL2 training dataset**		**Blind-test dataset**	
**length range**				
	**# regions**	**# residues**	**# regions**	**# residues**
1–3	483	1,044	791	1,440
4–15	758	5,650	1,012	7,343
16–30	148	3,118	151	3,173
31–100	154	8,039	50	2,236
>100	63	17,060	4	545

Total	1,606	34,911	2,008	14,737

**Table 2:** Prediction accuracies of the specialized predictors. The *per-chain *accuracies and standard errors were estimated via a 10-fold cross-validation procedure (see Performance evaluation). Using all 54 features, the two predictors were built as linear SVMs with parameter *C*, which represents the trade-off between training error and margin, set to 0.5 and 1, respectively. Default decision threshold of 0.5 was used for both predictors. *SN*_*S *_and *SN*_*L *_are *sensitivities*, or *true positive rates*, on *short *and *long *disordered regions, respectively. *SP *is *specificity*, or *true negative rate*, on ordered regions. *ACC*_*S *_is the overall accuracy for VSL2-S calculated as (*SN*_*S *_+ *SP*)/2, while *ACC*_*L *_is for VSL2-L as (*SN*_*L *_+ *SP*)/2.

	***W***_***in***_	***W***_***out***_	***SN***_***S***_	***SN***_***L***_	***SP***	***ACC***_***S***_/***ACC***_***L***_
	**15**	***5***	**82.0 ± 1.1**	**70.7 ± 1.9**	**81.5 ± 0.3**	**81.7 ± 0.6**
VSL2-S	15	31	56.5 ± 1.7	71.3 ± 2.3	89.1 ± 0.4	73.0 ± 0.8
	41	5	79.8 ± 1.3	72.8 ± 1.9	81.2 ± 0.4	80.5 ± 0.7
	41	31	68.8 ± 1.6	74.3 ± 2.2	85.3 ± 0.4	77.1 ± 0.8

	15	5	70.8 ± 1.4	78.6 ± 1.9	80.1 ± 0.6	79.4 ± 1.0
VSL2-L	15	31	53.4 ± 1.7	79.9 ± 2.2	84.9 ± 0.6	82.4 ± 1.1
	41	5	50.1 ± 1.7	80.9 ± 2.1	85.7 ± 0.5	83.3 ± 1.1
	**41**	**31**	**44.1 ± 1.8**	**82.1 ± 2.2**	**87.3 ± 0.5**	**84.7 ± 1.2**

**Table 3:** 20 features ranked on top using permutation test Z-scores. The features were ranked according to the absolute values of their permutation test Z-scores (see text). Name prefixes " *PSSM_*", "*freq_*", "*PHD_*" and " *PSI_*" denote PSI-BLAST profile based, amino acid frequency based, PHDsec prediction based, and PSIPRED prediction based features, respectively. *PSSM_41 *and *PSSM_42 *denote the two features derived from the last two columns of a PSI-BLAST profile (-Q option), i.e. *information per position *and *relative weight of gapless real matches to pseudocounts*.

	**VSL2-S**		**VSL2-L**	
**rank**				
	**feature name**	**Z-score**	**feature name**	**Z-score**
1	*freq_spacer*	166.3	*K*_2_*-entropy*	-265.6
2	*K*_2_*-entropy*	-91.4	*PSSM_P*	257.2
3	*PSI_C*	83.6	*PSSM_S*	228.3
4	*PHD_L*	81.2	*PSSM_K*	219.3
5	*PSSM_S*	78.3	*PSSM_E*	216.0
6	*PSSM_Q*	74.2	*PSI_C*	206.2
7	*hydrophobicity*	-70.5	*PSSM_Q*	189.3
8	*freq_H*	70.3	*hydrophobicity*	-183.5
9	*PSSM_E*	64.9	*PSSM_F*	-174.8
10	*PSSM_K*	64.7	*PHD_L*	170.8
11	*PSSM_42*	-63.7	*PSSM_T*	161.9
12	*flexibility*	58.4	*PSSM_L*	-156.9
13	*PSSM_N*	56.1	*PSSM_I*	-144.6
14	*PSSM_P*	51.7	*freq_P*	138.8
15	*PSSM_R*	46.3	*flexibility*	126.0
16	*PSSM_I*	-46.2	*freq_E*	122.0
17	*PSSM_D*	44.2	*PSSM_D*	99.3
18	*PSSM_V*	-42.9	*PSSM_M*	-97.2
19	*PSSM_H*	42.9	*freq_K*	96.2
20	*PSSM_41*	-40.9	*freq_I*	-90.0

**Table 4:** Choice of learning algorithms. Learning algorithms tested: LR – logistic regression; NN – neural network of 5 hidden nodes; NNE – bagging ensemble of 10 NNs; SVM/linear – linear support vector machine (inner-product kernel), with *C *= 0.5 for VSL2-S and *C *= 1 for VSL2-L; SVM/RBF – nonlinear support vector machine (radius-based-function kernel), with *C *= 2, *gamma = *2^-4^** for VSL2-S and *C *= 1, *gamma = *2**^-2 ^for VSL2-L. All 54 features were included to build the predictor models. The SVM parameters were selected by embedded 5-fold cross-validation (see Predictor model).

	**Learning algorithm**	***SN***_***S***_	***SN***_***L***_	***SP***	***ACC***_***S***_/***ACC***_***L***_
	LR	81.8 ± 1.1	70.2 ± 1.9	81.9 ± 0.3	81.8 ± 0.6
VSL2-S	NN	81.1 ± 1.1	68.6 ± 1.9	82.0 ± 0.3	81.5 ± 0.6
	NNE	81.5 ± 1.1	68.8 ± 2.0	83.3 ± 0.3	82.4 ± 0.6
	**SVM/linear**	**82.0 ± 1.1**	**70.7 ± 1.9**	**81.5 ± 0.3**	**81.7 ± 0.6**
	SVM/RBF	81.0 ± 1.1	70.0 ± 1.9	81.0 ± 0.3	81.0 ± 0.6

	LR	42.1 ± 1.8	80.3 ± 2.2	87.8 ± 0.5	84.0 ± 1.2
VSL2-L	NN	31.3 ± 1.7	76.6 ± 2.4	90.3 ± 0.5	83.4 ± 1.2
	NNE	31.9 ± 1.7	76.1 ± 2.4	91.9 ± 0.5	84.0 ± 1.2
	**SVM/linear**	**44.1 ± 1.8**	**82.1 ± 2.2**	**87.3 ± 0.6**	**84.7 ± 1.1**
	SVM/RBF	38.2 ± 1.7	80.2 ± 2.2	87.7 ± 0.6	83.9 ± 1.1

**Table 5:** Prediction accuracies of VSL2 predictors. The accuracies and standard errors were estimated via a 10-fold cross-validation procedure described (see Performance evaluation). The default decision threshold 0.5 was used for all four predictors. *SN *is the *overall sensitivity*, or *true positive rate*, on all disordered regions, and *ACC *is the *overall accuracy *calculated as *(SN + SP)/*2.

	***SN***	***SP***	***ACC***	***SN*_*S*_**	***SN*_*L*_**
VSL2-S	79.8 ± 1.0	81.5 ± 0.3	80.7 ± 0.5	82.0 ± 1.1	70.7 ± 1.9
VSL2-L	54.2 ± 1.6	87.3 ± 0.5	70.7 ± 0.8	44.1 ± 1.8	82.1 ± 2.2
**VSL2-M1**	**82.3 ± 1.1**	**81.0 ± 0.5**	**81.6 ± 0.5**	**81.3 ± 1.2**	**82.3 ± 1.8**
VSL2-M2	82.1 ± 1.0	80.7 ± 0.5	81.4 ± 0.5	81.1 ± 1.2	81.8 ± 1.8

(a) *per-chain*					

	***SN***	***SP***	***ACC***	***SN***_***S***_	***SN***_***L***_

VSL2-S	75.2 ± 2.5	81.3 ± 0.3	78.3 ± 1.3	78.1 ± 1.3	74.1 ± 3.5
VSL2-L	74.7 ± 2.9	89.0 ± 0.5	81.9 ± 1.6	47.3 ± 2.0	84.4 ± 3.1
**VSL2-M1**	**82.9 ± 2.1**	**81.6 ± 0.4**	**82.3 ± 1.1**	**77.6 ± 1.4**	**84.7 ± 2.7**
VSL2-M2	82.8 ± 2.1	81.6 ± 0.5	82.2 ± 1.1	78.3 ± 1.3	84.3 ± 2.6

(b) *per-residue*					

**Table 6:** Importance of computationally expensive features. A "+" mark indicates if a feature set was included in predictor construction. The feature sets were: **AA **-26 features directly calculated from the amino acid sequence; **PSSM **– 22 features as average PSI-BLAST profiles over the input window; **PHD **– 3 features as average secondary structure prediction scores by the PHDsec predictor; **PSI **– 3 features as average secondary structure prediction scores by the PSIPRED predictor. **F **is the total number of features included. The 1^st^, 5^th^, and 8^th ^rows correspond to VSL2B, VSL2P, and VSL2, respectively. All accuracies are *per-chain *accuracies.

**AA**	**PSSM**	**PHD**	**PSI**	**F**	***SN***	***SP***	***ACC***	***SN*_*S*_**	***SN*_*L*_**
+				**26**	**77.3 ± 1.1**	**79.9 ± 0.4**	**78.6 ± 0.6**	**75.8 ± 1.3**	**78.2 ± 1.9**
+		+		29	77.9 ± 1.1	80.4 ± 0.4	79.1 ± 0.6	76.6 ± 1.3	78.5 ± 2.0
+			+	29	79.9 ± 1.1	79.9 ± 0.4	79.9 ± 0.5	78.7 ± 1.3	79.9 ± 2.0
+		+	+	32	79.8 ± 1.1	80.3 ± 0.4	80.1 ± 0.5	78.6 ± 1.2	79.8 ± 2.0
+	+			**48**	**81.0 ± 1.0**	**80.4 ± 0.5**	**80.7 ± 0.6**	**79.8 ± 1.2**	**80.9 ± 1.9**
+	+	+		51	81.3 ± 1.0	81.0 ± 0.5	81.2 ± 0.5	80.0 ± 1.2	81.4 ± 1.8
+	+		+	51	82.3 ± 1.0	80.5 ± 0.5	81.4 ± 0.5	81.1 ± 1.2	81.9 ± 1.9
+	+	+	+	**54**	**82.3 ± 1.1**	**81.0 ± 0.5**	**81.6 ± 0.5**	**81.3 ± 1.2**	**82.3 ± 1.8**

**Table 7:** Comparison to other protein disorder predictors over VSL2 training dataset via 10-fold cross-validation. Predictions by VSL2B, VSL2P and VSL2 were made via the 10-fold cross-validation procedure, while other predictors were applied to the 1,327 sequences directly. Default thresholds were used for all predictors, e.g. 0.5 for VL-XT, VL3-E, VSL1, VSL2B, VSL2P and VSL2. Also shown are the areas under ROC curves (*AUC*) in Figure 5.

	***SN***	***SP***	***ACC***	***SN***_***S***_	***SN***_***L***_	***AUC***
VL-XT	58.6 ± 1.3	78.6 ± 0.4	68.6 ± 0.7	56.3 ± 1.6	63.5 ± 2.1	75.7 ± 0.8
VL3-E	38.7 ± 1.6	92.7 ± 0.5	65.7 ± 0.8	23.5 ± 1.6	82.5 ± 2.2	76.0 ± 0.8
DisEMBL	31.4 ± 1.4	97.8 ± 0.1	64.6 ± 0.7	30.5 ± 1.6	32.8 ± 2.2	84.5 ± 0.6
RONN	45.8 ± 1.4	87.0 ± 0.4	66.4 ± 0.7	39.6 ± 1.7	63.0 ± 2.3	72.2 ± 0.9
DISOPRED2	56.9 ± 1.3	94.1 ± 0.2	75.5 ± 0.7	56.5 ± 1.6	55.1 ± 2.4	85.7 ± 0.6
**VSL1**	**79.0 ± 1.1**	**85.3 ± 0.4**	**82.2 ± 0.6**	**78.0 ± 1.3**	**78.1 ± 2.0**	**89.9 ± 0.6**
VSL2B	77.3 ± 1.1	79.9 ± 0.4	78.6 ± 0.6	75.8 ± 1.3	78.2 ± 1.9	86.0 ± 0.6
VSL2P	81.0 ± 1.0	80.4 ± 0.5	80.7 ± 0.6	79.8 ± 1.2	80.9 ± 1.9	88.0 ± 0.6
**VSL2**	**82.3 ± 1.1**	**81.0 ± 0.5**	**81.6 ± 0.5**	**81.3 ± 1.2**	**82.3 ± 1.8**	**89.2 ± 0.5**

(a) *per-chain*						

	***SN***	***SP***	***ACC***	***SN***_***S***_	***SN***_***L***_	***AUC***

VL-XT	58.9 ± 1.7	79.2 ± 0.3	69.0 ± 0.9	51.4 ± 1.7	61.6 ± 2.2	75.7 ± 1.2
VL3-E	70.4 ± 3.1	94.5 ± 0.4	82.5 ± 1.6	27.2 ± 1.9	85.7 ± 2.7	90.9 ± 1.0
DisEMBL	32.5 ± 2.0	98.2 ± 0.1	65.4 ± 1.0	28.7 ± 1.5	33.9 ± 2.7	80.0 ± 1.1
RONN	61.1 ± 2.9	87.6 ± 0.3	74.4 ± 1.5	42.7 ± 1.9	67.6 ± 3.3	81.5 ± 1.5
DISOPRED2	60.2 ± 3.7	95.1 ± 0.2	77.6 ± 1.8	50.1 ± 1.7	63.7 ± 4.6	87.7 ± 1.2
**VSL1**	**78.1 ± 2.3**	**86.7 ± 0.3**	**82.4 ± 1.2**	**71.4 ± 1.5**	**80.4 ± 2.9**	**90.3 ± 1.0**
VSL2B	77.0 ± 2.2	81.5 ± 0.3	79.3 ± 1.1	67.6 ± 1.6	80.4 ± 2.7	87.1 ± 1.2
VSL2P	81.7 ± 2.2	82.2 ± 0.4	81.9 ± 1.1	75.6 ± 1.5	83.8 ± 2.7	89.8 ± 1.2
**VSL2**	**82.9 ± 2.1**	**81.6 ± 0.4**	**82.3 ± 1.1**	**77.6 ± 1.4**	**84.7 ± 2.7**	**90.5 ± 1.1**

(b) *per-residue*						

**Table 8:** Comparison to other protein disorder predictors over a blind-test set. The non-redundant blind-test set contained 1,304 recent PDB chains that were unrelated to any VSL2 training sequence. The three VSL2 predictors were re-trained with *all *1,327 training sequences. The prediction accuracies were calculated at default thresholds, e.g. 0.5 for VL-XT, VL3-E, VSL1, VSL2B, VSL2P and VSL2.

	*****SN*****	***SP***	***ACC***	***SN***_*S*_	***SN***_*L*_	***AUC***
VL-XT	56.0	77.9	67.0	55.8	53.0	74.4
VL3-E	28.4	91.4	59.9	26.8	59.7	69.2
DisEMBL	25.9	97.9	61.9	25.7	26.5	83.9
RONN	34.7	88.8	61.8	33.6	56.4	67.2
DISOPRED2	53.8	94.7	74.2	53.5	48.6	84.6
**VSL1**	**75.6**	**85.8**	**80.7**	**76.1**	**63.6**	**88.5**
VSL2B	73.7	80.8	77.3	74.2	63.1	84.1
VSL2P	78.4	80.4	79.4	78.5	71.6	86.7
**VSL2**	**79.4**	**81.4**	**80.4**	**79.6**	**73.7**	**87.5**

(a) *per-chain*						

	***SN***	***SP***	***ACC***	***SN***_***S***_	***SN***_*L*_	***AUC***

VL-XT	54.2	79.2	66.7	54.7	52.3	74.0
VL3-E	36.5	93.9	65.2	30.5	59.2	75.9
DisEMBL	25.3	98.3	61.8	25.4	24.7	80.1
RONN	41.6	89.1	65.4	39.1	51.2	71.3
DISOPRED2	47.8	95.6	71.7	48.7	44.6	83.4
**VSL1**	**68.3**	**87.4**	**77.9**	**70.9**	**58.3**	**86.2**
VSL2B	67.1	82.6	74.8	68.9	60.3	82.0
VSL2P	72.7	82.5	77.6	74.2	67.1	85.3
**VSL2**	**74.7**	**82.8**	**78.7**	**76.1**	**69.4**	**86.3**

(b) *per-residue*						
